# M. Bricheteau on the Antagonism of Ague, and of Pulmonary Consumption

**Published:** 1846-04

**Authors:** 


*M. Bricheteau on the Antagonism of Ague, and of Pulmonary Consumption.*—This question has been much discussed of late by French medical practitioners, as our readers are well aware. M. Bricheteau, physician to the " Hôpital Necker," analyzes the various communications that have appeared on the subject, including documents from different parts of Algeria, from Bourdeaux, Strasbourg, Lyons, the department of the Ain, Rochefort, Rome, &c.,—all localities in which intermittent fever is rife,—and appears to come to the conclusion that there cannot be said to be antagonism between the two diseases—that is, exclusion of the one by the other; although the circumstances which favour the development of intermittents may be, and in all probability are, unfavourable to the development of phthisis. M. Bricheteau thus concludes his remarks :—

" Although, on examining the etiology of these diseases, we do not find incompatibility between the causes of phthisis and intermittent fevers, it is impossible not to recognise, either in the climate of marshy districts, or in the influence of marshy miasmata over the economy, conditions favourable to tubercular patients. Our knowledge of this fact is to be referred to the authors of the labours which we have enumerated. But instead of calling to our assistance some obscure antagonizing tendencies, would it not be possible to account for this kind of prophylaxy, by attributing it to the moist uniform heat which reigns in some marshy districts, and which, by favouring the development of fever, may impede that of pulmonary tuberculization. Does not this appear proved by what takes place at Strasbourg, where the climate being both damp and cold, the town is ravaged by intermittent fever and by phthisis; whereas the more southern departments of L'Ain, La Niévra, Le Var, &c., are decimated by intermittent fevers, but offer very few phthisical patients? We may also add, that it is impossible to deny that in all countries intermittent fevers preserve from other affections. The Dutch appear to be aware of this fact, as Boerhaave informs us, that they are in the habit of congratulating themselves on the return of their fevers. The same Boerhaave, along with Hoffmann, Lancisi, and Sydenham, thought that intermittent fevers freed us from various diseases, and even predisposed to longevity: 'Febres intermittentes, nisi malignæ, ad longevitatem disponunt, et depurant ab inveteratis malis.' Some recent writers think that typhus fever is rarely met with in countries ravaged by endemic intermittents."—*Ibid.*

